# Molecular characterization of freshwater snails in the genus *Bulinus*: a role for barcodes?

**DOI:** 10.1186/1756-3305-1-15

**Published:** 2008-06-10

**Authors:** Richard A Kane, J Russell Stothard, Aidan M Emery, David Rollinson

**Affiliations:** 1 Department of Zoology, Natural History Museum, South Kensington, Cromwell Road, London SW7 5BD, UK

## Abstract

**Background:**

Reliable and consistent methods are required for the identification and classification of freshwater snails belonging to the genus *Bulinus *(Gastropoda, Planorbidae) which act as intermediate hosts for schistosomes of both medical and veterinary importance. The current project worked towards two main objectives, the development of a cost effective, simple screening method for the routine identification of *Bulinus *isolates and the use of resultant sequencing data to produce a model of relationships within the group.

**Results:**

Phylogenetic analysis of the DNA sequence for a large section (1009 bp) of the mitochondrial gene cytochrome oxidase subunit 1 (*cox1*) for isolates of *Bulinus *demonstrated superior resolution over that employing the second internal transcribed spacer *(its2*) of the ribosomal gene complex. Removal of transitional substitutions within *cox1 *because of saturation effects still allowed identification of snails at species group level. Within groups, some species could be identified with ease but there were regions where the high degree of molecular diversity meant that clear identification of species was problematic, this was particularly so within the *B. africanus *group.

**Conclusion:**

The sequence diversity within *cox1 *is such that a barcoding approach may offer the best method for characterization of populations and species within the genus from different geographical locations. The study has confirmed the definition of some accepted species within the species groups but additionally has revealed some unrecognized isolates which underlines the need to use molecular markers in addition to more traditional methods of identification. A barcoding approach based on part of the *cox1 *gene as defined by the Folmer primers is proposed.

## Background

**Figure 1:**
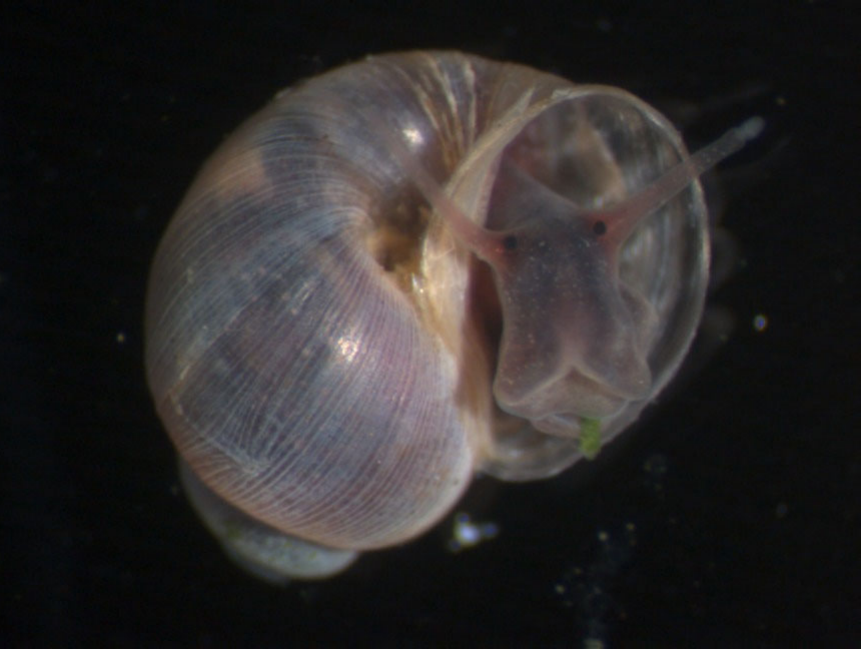
***Bulinus wrighti***. *Bulinus wrighti *an intermediate host of *Schistosoma haematobium *in South Yemen.

Freshwater snails belonging to the genus *Bulinus *act as intermediate hosts in the life cycle of the widespread and debilitating parasitic disease schistosomiasis in Africa, Madagascar and adjacent regions (see Fig. 1). Schistosome species within the *S. haematobium *group which depend on snails from *Bulinus *for transmission include three human pathogens (*S. haematobium*, *S. intercalatum *and *S. guiniensis*) and five others which may infect wild and domestic ruminants (*S. bovis*, *S. curassoni*, *S. mattheei*, *S. leiperi *and *S. margrebowiei*). The relationship and interaction between schistosomes and snails is very specific and compatibility may differ over quite small geographical ranges [1]. A reliable taxonomy of the genus *Bulinus *is a fundamental prerequisite for understanding the epidemiology of this disease.

The thirty six species within the genus have been placed into four species 'groups' [2]; the *B. africanus *group, *B. forskalii *group, *B. reticulatus *group and the *B. truncatus/tropicus *complex. For the most part, species have been classified on the basis of their morphology although, in recent decades, the study of ploidy [3], allozymes [4-6] and DNA methods [7-13] have all played an increasing role in species discrimination. Morphological characters, whilst adequate to allocate a specimen to a species group are sometimes unreliable when used to classify at higher resolution [8,10] especially within the *B. africanus *group. Consequently, there is a requirement for a robust system of identification and classification to supplement more traditional approaches. The data presented here represent initial steps towards achieving consistency and uniformity in the identification process. Nuclear (*its2*) and mitochondrial (*cox1*) sequences are compared with respect to their ability to resolve species and species group relationships within *Bulinus *and *cox1 *is used to screen 81 isolates and place them in a phylogenetic context.

## Methods

### Samples

The available samples selected for study represented as diverse a group as possible within the genus *Bulinus *and comprised of both collected and donated material that took the form of specimens from the field and maintained cultures which were stored in liquid nitrogen, ethanol or used fresh (Table 1). Those specimens preserved in ethanol were left in TE buffer pH 7.4 overnight in order to perfuse out any remaining alcohol from within the tissue that might interfere with subsequent extraction techniques.

**Table 1:** Isolates included in the study with key to alphanumeric identifiers used in the Figures.

**Label on tree**	**Field collected (F) or Lab culture (L)**	**Putative identification**	**Sample origin**	**GPS Co-ordinate where available**	**Accession numbers**
A1	F	*B. globosus*	Pemba Island, Tanzania	S05 24 626/E39 43 756	AM921823
A2	F	*B. globosus*	Ngwachani school, Pemba Island, Tanzania	S05 19 364/E39 44 517	AM921826
A3	F	*B. globosus*	Chan-jamjawiri, Pemba Island, Tanzania	S05 18 094/E39 45 192	AM921828
A4	F	*B. globosus*	Machengwe, Pemba Island, Tanzania	S05 04 957/E39 45 814	AM921829
A5	F	*B. globosus*	Kimbuni, Pemba Island, Tanzania	S05 21 231/E39 42 841	AM921830
A6	F	*B. globosus*	Road to Mtangani, Pemba Island, Tanzania	S05 21 525/E39 46 122	AM921820
A7	F	*B. globosus*	Road to Mtangani, Pemba Island, Tanzania	S05 21 600/E39 46 125	AM921825
A8	F	*B. globosus*	Ngwachani school, Pemba Island, Tanzania	S05 19 364/E39 44 517	AM921827
A9	F	*B. globosus*	Pietermaritzburg, South Africa (Prof. K. N. De Kock)		AM286289
A10	F	*B. globosus*	Pietermaritzburg, South Africa (Prof. K. N. De Kock)		AM286290
A11	F	*B. globosus*	Tiengre stream, Kisumu, West Kenya (via DBL)		AM286285
A12	F	*B. globosus*	Tiengre stream, Kisumu, West Kenya (via DBL)		AM286284
A13	F	*B. globosus*	Kandaria dam, Kisumu, West Kenya (via DBL)		AM286286
A14	F	*B. globosus*	Ipogoro, Iringa, Tanzania		AM286288
A15	F	*B. globosus*	Lugufu, Tanzania (Dr E. Michel)	S05 05 371/E30 11 689	AM286287
A16	F	*B. globosus*	IRDC farm, Iringa, Tanzania (Dr S. Walker)	S07 46 290/E35 45 590	AM921821
A17	F	*B. globosus*	IRDC farm, Iringa, Tanzania (Dr S. Walker)	S07 46 285/E35 45 355	AM921824
A18	F	*B. globosus*	Moyo, Uganda	N03 28 219/E31 55 360	AM286291
A19	F	*B. globosus*	Moyo, Uganda	N03 28 219/E31 55 360	AM921843
A20	F	*B. globosus*	Moyo, Uganda	N03 28 219/E31 55 360	AM921851
A21	F	*B. globosus*	Kachetu, East Kenya	S03 54 350/E39 32 250	AM921847
A22	F	*B. globosus*	Mwamduli, East Kenya	S03 54 350/E39 31 470	AM921850
A23	F	*B. globosus*	Kinyasini, Unguja Island, Tanzania	S05 58 180/E39 18 573	AM286292
A24	F	*B. globosus*	Kinango, East Kenya	S04 05 263/E39 18 721	AM921845
A25	F	*B. globosus*	Kinango, East Kenya	S04 05 263/E39 18 721	AM921844
A26	F	*B. globosus*	Kinyasini, Unguja, Island, Tanzania	S05 58 180/E39 18 573	AM921839
A27	F	*B. globosus*	Kinyasini, Unguja, Island, Tanzania	S05 58 180/E39 18 573	AM921840
A28	F	*B. africanus*	Isipingo, Durban, South Africa, (Prof. C. Appleton)	S29 58 584/E30 55 503	AM286295
A29	F	*B. africanus*	Isipingo, Durban, South Africa, (Prof. C. Appleton)	S29 58 584/E30 55 503	AM286296
A30	F	*B. globosus*	Mogtedo barrage, Burkina Faso	N12 18 388/W00 49 670	AM286293
A31	F	*B. globosus*	Tondia, Niger	N14 28 348/W01 05 635	AM286294
A32	F	*B. globosus*	Thiekeene Hulle, Senegal		AM921808
A33	F	*Bulinus *sp	ADC farm, Kisumu, West Kenya (via DBL)		AM286297
A34	F	*Bulinus *sp	Lake Sagara, Tanzania (Dr E. Michel)	S05 15 084/E31 05 111	AM286298
A35	F	*B. nasutus productus*	Road to Cawente, Uganda	N01 49 341/E33 32 235	AM921815
A36	F	*B. nasutus productus*	Road to Cawente, Uganda	N01 49 341/E33 32 235	AM921816
A37	F	*B. nasutus productus*	Ihayabuyaga, Tanzania		AM286300
A38	F	*B. nasutus productus*	Njombe Rujewa, Tanzania, (Dr S. Walker)		AM921833
A39	F	*B. nasutus productus*	Ihayabuyaga, Tanzania		AM286301
A40	F	*B. nasutus productus*	Kahangara, Tanzania		AM286302
A41	F	*B. nasutus nasutus*	Vitonguji, Pemba Island, Tanzania	S05 14 027/E39 49 706	AM921810
A42	F	*B. nasutus nasutus*	Pujini Kikweche, Pemba Island, Tanzania	S05 18 843/E39 47 863	AM921822
A43	F	*B. nasutus nasutus*	Pujini, Pemba Island, Tanzania	S05 18 988/E39 48 667	AM921813
A44	F	*B. nasutus nasutus*	Vitonguji, Pemba Island, Tanzania	S05 14 027/E39 49 706	AM921809
A45	F	*B. nasutus nasutus*	Muyuni, Unguja, Tanzania	S06 22 707/E39 27 849	AM286299
A46	F	*B. nasutus nasutus*	Pemba Island, Tanzania	S05 10 272/E39 49 319	AM921812
A47	F	*B. nasutus nasutus*	Pemba Island, Tanzania	S05 10 272/E39 49 319	AM921811
A48	F	*B. nasutus nasutus*	Mafia Island, Tanzania	S07 50 838/E39 47 354	AM921831
A49	F	*B. nasutus nasutus*	Bovo, East Kenya	S04 28 054/E39 28 108	AM921849
A50	F	*B. nasutus nasutus*	Nimbodze, East Kenya	S04 28 317/E39 27 092	AM921841
A51	F	*B. nasutus nasutus*	Nimbodze, East Kenya	S04 28 323/E39 27 098	AM921846
F52	F	*Bulinus *sp	Road to Cawente, Uganda	N01 49 341/E33 32 235	AM921819
F53	F	*B. camerunensis*	Lake Barombi, Kotto, Cameroon		AM286309
F54	F	*B. forskalii*	Mogtedo barrage, Burkina Faso	N12 18 388 W00 49 670	AM286310
F55	F	*B. forskalii*	Satoni, Niger		AM286308
F56	L	*B. forskalii*	Dakar, Senegal		AM286307
F57	L	*B. forskalii*	City of São Tomé, São Tomé Island		AM286305
F58	L	*B. forskalii*	Quifangondo, Province of Bengo, Angola		AM286306
F59	F	*Bulinus *sp	Pemba Island, Tanzania	S04 55 682/E39 44 271	AM921832
F60	F	*Bulinus *sp	Kamwiju Kaloleni, East Kenya		AM921848
F61	L	*B. cernicus*	Mont Oreb, Mauritius		AM286303
F62	L	*B. cernicus*	Perebere, Mauritius		AM286304
F63	F	*B. barthi*	Kangagani, Pemba Island, Tanzania	S05 09 911/E39 49 527	AM921818
F64	F	*B. barthi*	Kanga swamp, Mafia Island, Tanzania	S07 43 358/E39 51 505	AM921814
F65	F	*B. barthi*	Kanga swamp, Mafia Island, Tanzania	S07 43 362/E39 51 505	AM921817
R66	L	*B. wrighti*	Oman (via Perpignan)		AM286318
T67	L	*B. natalensis*	Lake Sibaya, South Africa		AM286311
T68	L	*B. natalensis*	Lake Sibaya, South Africa		AM921835
T69	L	*B. natalensis*	Lake Sibaya, South Africa		AM921836
T70	F	*B. tropicus*	Njombe Kibena, Tanzania, (Dr S. Walker)	S09 12 229/E34 47 041	AM921842
T71	F	*B. tropicus*	Njombe Kibena, Tanzania, (Dr S. Walker)	S09 12 229/E34 47 041	AM921834
T72	F	*B. tropicus*	Njombe Kibena, Tanzania, (Dr S. Walker)	S09 12 229/E34 47 041	AM921837
T73	F	*B. nyassanus*	Kasankha, Monkey Bay, Lake Malawi	Transect line north: E07 00 595/N84 38 260 Transect line south: E07 00 617/N84 38 277	AM921838
T74	F	*B. truncatus*	Nyanguge, Tanzania		AM286313
T75	F	*B. truncatus*	Posada, Sardinia (Prof. Marco Curini Galletti & Dr D.T.J. Littlewood)	N40 38 092/E09 40 522	AM286312
T76	L	*B. truncatus*	Mondego River, Coimbra, Portugal (Prof. M.A. Gracio)		AM286314
T77	F	*B. truncatus*	Mbane, Senegal		AM921806
T78	F	*B. truncatus*	Bouton Batt, Senegal		AM921807
T79	F	*B. truncatus*	Mogtedo barrage, Burkina Faso	N12 18 388/W00 49 670	AM286315
T80	F	*B. truncatus*	Satoni, Niger	N14 26 671/E01 07 257	AM286316
T81	F	*B. truncatus*	Satoni, Niger	N14 26 685/E01 07 316	AM286317
*B. glabrata*		*B. glabrata*	Brazil		AY380531

### Extraction of genomic/mitochondrial DNA

Total genomic DNA was extracted from whole snail tissue in a manner similar to that outlined by Stothard *et al *[7] with minor modification. Snail tissue was homogenized in lysis buffer (100 mM Tris, 1.4 M NaCl, 16 mM EDTA, 2% hexadecyltrimethylammonium bromide [CTAB]). To this was added 20 μl of proteinase K (20 mg/ml) and the whole mixed in a rotisserie incubator at 55°C for 1.5 to 2.0 hours. Subsequently, an equal volume of chloroform/isoamyl alcohol (24:1) was added to the digest and gently mixed. Tubes containing the digest were then spun at 13,000 rpm for 10 minutes. The upper aqueous layer was removed using 'wide bore' pipette tips and nucleic acids were precipitated in 'Analar' grade absolute ethanol. Following precipitation for 15 minutes at -20°C, the DNA was centrifuged again at 13,000 rpm to form a pellet. The absolute ethanol was removed and the pellet washed in 70% ethanol before a final centrifugation at 13,000 rpm. The ethanol was then discarded and the pellet dried in a dry heating block at 90°C for 5 minutes before dissolution in an appropriate amount of purified water.

### Amplification of *cox1* and *its2* fragments

**Figure 2:**
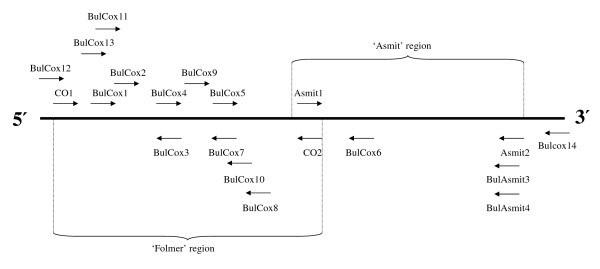
**Locations of primers used for the *cox1 *fragment**. Locations of primers used for *cox1 *PCR amplification and sequencing. Folmer and Asmit regions are indicated. Not to scale.

The partial *cox1 *fragment was amplified in one, two or more sections from the mitochondrial component of extracted total genomic DNA using the polymerase chain reaction (PCR) and various combinations of the primers that are shown in Table 2 (see additional file 1, for PCR and sequencing primers that proved successful with particular isolates and Fig. 2 for approximate primer locations). Amplicons of *its2 *were generated in a single section using two primers ETTS1 and ETTS10 (see Table 2). Either an Applied Biosystems GeneAmp PCR System 2400 or 2700 thermal cycler were used throughout the project in combination with GE Healthcare 'Ready-To-Go' PCR beads. Upon reconstitution with an appropriate volume of template, primer and pure water to a total of 25 μl, each dissolved bead forms a solution containing 200 μM of each dNTP, 10 mM Tris-HCl, 50 mM KCl and 1.5 mM of MgCl_2_. Cycling conditions for both *cox1 *and *its2 *PCR reactions were as follows: one cycle of 94°C for 5 min, 45 cycles of 94°C for 15 sec, 40°C for 30 sec and 72°C for 45 sec (in the case of *cox1 *this was increased to 1 min for amplicons over 1000 bp) and a final single cycle of 72°C for 7 min. PCR fragments were separated on a 1% agarose gel and bands were excised using a scalpel blade. The recovered DNA was purified for sequencing using a QIAquick Gel Extraction Kit (Qiagen). Following quantification and a check for purity with a Nanodrop ND-1000 Spectrophotometer (Nanodrop Technologies Inc), sequencing reactions were performed directly on each PCR product using an Applied Biosystems Big Dye Kit version 1.1 and run on an Applied Biosystems 3730 DNA Analyzer. Resultant electropherograms were checked and *cox1/its2 *sequences edited using Sequencher 4.6 software (Gene Codes Corporation).

**Table 2:** Primers used for PCR amplification and sequencing

**Primer name**	**Cytochrome oxidase subunit 1 – primer sequence**	**Forward or Reverse**	**Source**
Asmit1 (AT1)	5' TTT TTT GGG CAT CCT GAG GTT TAT 3'	Forward	[26]
Asmit2 (AT2)	5' TAA AGA AAG AAC ATA ATG AAA ATG 3'	Reverse	[26]
CO1 (LC1490)	5' GGT CAA CAA ATC ATA AAG ATA TTG G 3'	Forward	[18]
CO2 (HCO2198)	5' TAA ACT TCA GGG TGA CCA AAA AAT CA 3'	Reverse	[18]
BulCox1 (BC1)	5' TTT TTG GWG TTT GAT GTG G 3'	Forward	NHM – current project
BulCox2 (BC2)	5' TGT GGT CTG GTA GGW ACC GG 3'	Forward	NHM – current project
BulCox3 (BC3)	5' CGT GGA AAW CTT ATA TCW GGW GC 3'	Reverse	NHM – current project
BulCox4 (BC4)	5' GCW CCW GAT ATA AGW TTT CCA CG 3'	Forward	NHM – current project
BulCox5 (BC5)	5' CCT TTA AGA GGN CCT ATT GC 3'	Forward	NHM – current project
BulCox6 (BC6)	5' CAA TAA ACC CTA AAA TYC C 3'	Reverse	NHM – current project
BulCox7 (BC7)	5' GCA ATA GGT CCT CTT AAA GG 3'	Reverse	NHM – current project
BulCox8 (BC8)	5' GTA ATA AAA TTA ATW GCA CCT AAA A 3'	Reverse	NHM – current project
BulCox9 (BC9)	5' CCW CCT TCA TTT ATT TT 3'	Forward	NHM – current project
BulCox10 (BC10)	5' GCT AAA TGT AAA G 3'	Reverse	NHM – current project
BulCox11 (BC11)	5' TTT TGG DRT YTG RTG YGG 3'	Forward	NHM – current project
BulCox12 (BC12)	5' GCG TTG ACT CTT TTC AAC 3'	Forward	NHM – current project
BulCox13 (BC13)	5' CWT TRT AYW TAA TTT TTG G 3'	Forward	NHM – current project
BulCox14 (BC14)	5' GGA AAT CAG TAM AYA AAA CCA GC 3'	Reverse	NHM – current project
BulAsmit3 (BAT3)	5' CAT AAT GAA AAT GAG CAA CTA C 3'	Reverse	NHM – current project
BulAsmit4 (BAT4)	5' CAT AAT GAA AAT GAG C 3'	Reverse	NHM – current project
**Primer name**	**Second internal transcribed spacer of the ribosomal gene complex – primer sequence**	**Forward or Reverse**	**Source**
ETTS1	5' TGC TTA AGT TCA GCG GGT 3'	Reverse	[27]
ETTS10	5' GCA TAC TGC TTT GAA CAT CG 3'	Forward	[27]

### Phylogenetic analysis of sequence data

Following basic editing and analysis of compiled *cox1 *and *its2 *sequences on Sequencher 4.6, the sequences were used to perform BLAST searches [14] via the National Center for Biotechnology Information against GenBank and EMBL sequence databases in order to ensure that parasitic and other potential contaminant sequences had not been obtained in error. Sequences were then aligned and analysed using MEGA 3.1 [15] where alignment was undertaken using Clustal W [16]. The *cox1 *data for all taxa were analysed solely as nucleotides and subject to analysis by both Neighbour-Joining and Minimum Evolution methods using Kimura's 2 parameter model (K2P) for pair-wise distance calculations as this accommodates the difference in the rate of accumulation between transitions and transversions. The Minimum Evolution algorithm employed Close-Neighbour-Interchange (CNI – level 1) to explore the most optimal topology with the initial, temporary tree obtained by Neighbour-Joining. All gaps were deleted from the dataset using the 'complete deletion' option in MEGA 3.1 and the invertebrate mitochondrial code was used throughout. Nucleotide sequence data for *its2 *was analysed in a similar manner except that p-distance was employed instead of K2P.

The different forms of analysis were subject to bootstrapping (1000 repeats) as a means of testing the reliability of individual branches within the generated tree. *Biomphalaria glabrata *was used as an outgroup taxon upon which to root the structures. Sequence saturation for *cox1 *was visualized graphically, using the program DAMBE [17] that allows the number of differences between isolates or species in terms of transitional and transversional substitutions to be plotted against pair-wise distance values.

Sequences have been submitted to the EMBL database and have accession numbers [EMBL:AM286284 to AM286318, EMBL:AM921806 to AM921851 (*cox1*) and EMBL:AM921961 to AM921990 (*its2*)].

## Results

### Analysis of *its2* sequence data

**Figure 3:**
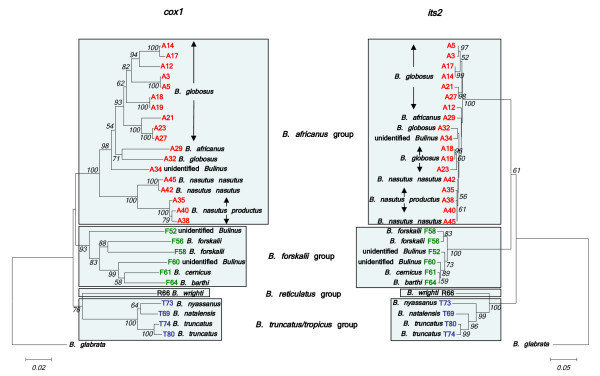
**Comparison of *cox1 *and *its2 *Neighbour-Joining trees for *Bulinus***. Comparison of Neighbour-Joining trees for *cox1 *and *its2 *data (*cox1 *– 1010 sites and *its2 *– 394 sites). Kimura 2-parameter distance has been used for *cox1 *and p-distance for *its2*. Substitutions include both transitions and transversions and 1000 bootstrap replicates have been performed. Bootstrap values below 50 are not shown. *B. africanus *group isolates are shown in red, *B. forskalii *in green, *B. reticulatus *in black and *B. truncatus/tropicu*s in blue. See Table 1 for origins of isolates.

Twenty nine samples were selected for sequencing of an amplicon containing the 3' end of the 5.8S gene and the entire *its2 *region. This was undertaken in order to compare the phylogenetic signal of a nuclear marker with that of *cox1 *(see Fig. 3). Both loci were able to discriminate the 4 species groups in a Neighbour-Joining tree. The *B. forskalii*, *B. reticulatus *and *B. truncatus/tropicus *species groups resolved well, the main point of difference being F52 which in the *its2 *tree had closer affinity with the East African *B. forskalii *group snails. The *B. africanus *group within the *its2 *tree had short branch lengths with poor resolution and bootstrap values. Clear discrimination between *B. nasutus nasutus *and *B. nasutus productus *was not possible using the *its2 *data and sequence differences in this region are unlikely to be of value for the detection of hybridization events among isolates with overlapping geographic ranges. The *its2 *sequence data for *B. wrighti *were unusual in having two unique insertions relative to other *Bulinus *isolates, a short 16 bp insertion between fragment positions 219 and 234 and a larger 63 bp insertion between positions 248 and 310.

### Analysis of *cox1* sequence data

**Figure 4:**
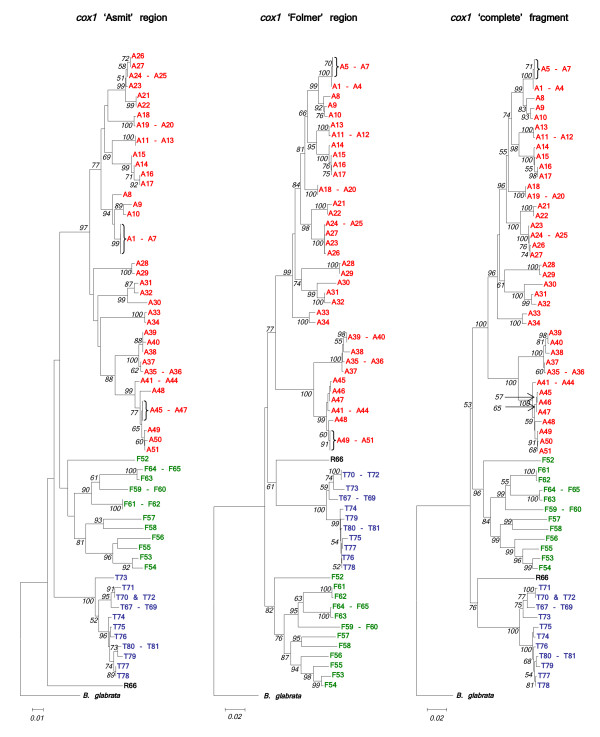
**Three regions of the *cox1 *fragment compared using Neighbour-Joining trees**. Comparison of Neighbour-Joining trees using Kimura 2-parameter distance for 'Asmit' (final 387 sites), 'Folmer' (first 644 sites) and 'complete' (1009 sites) sections of the *cox1 *gene. Substitutions include both transitions and transversions and 1000 bootstrap replicates have been performed. Bootstrap values below 50 are not shown. *B. africanus *group isolates are shown in red, *B. forskalii *in green, *B. reticulatus *in black and *B. truncatus/tropicu*s in blue. See Table 1 for origins of isolates.

**Figure 5:**
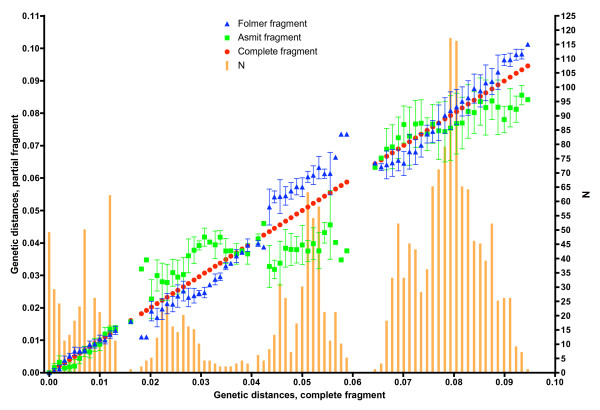
**Comparison of regional *cox1 *genetic distances using transversions only**. Plot showing mean, pair-wise genetic distance values of the 'Asmit' and 'Folmer' regions using transversions only as compared with the corresponding values for the complete fragment. Number of pairs contributing to each value is shown graphically.

**Figure 6:**
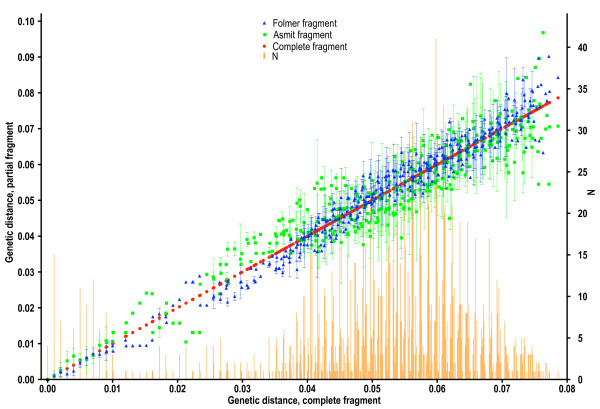
**Comparison of regional *cox1 *genetic distances using transitions only**. Plot showing mean, pair-wise genetic distance values of the 'Asmit' and 'Folmer' regions using transitions only as compared with the corresponding values for the complete fragment. Number of pairs contributing to each value is shown graphically.

**Figure 7:**
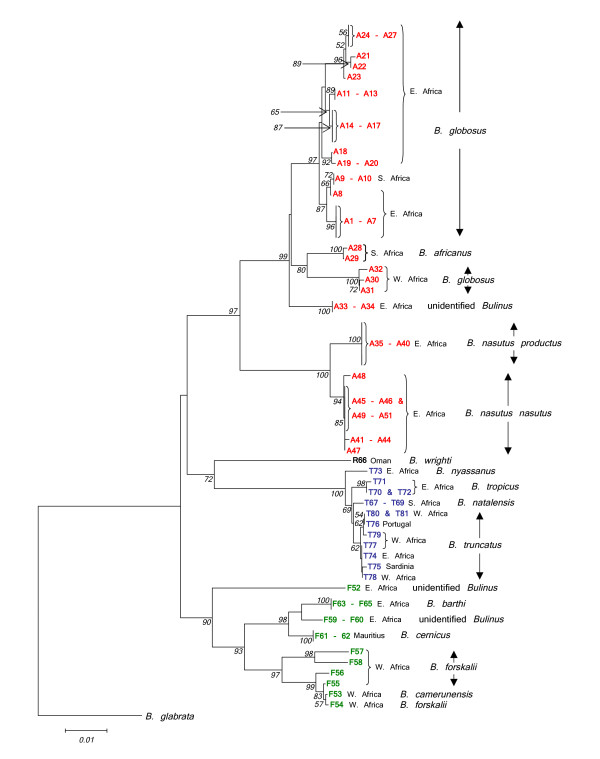
**Neighbour-Joining tree for *Bulinus *isolates using the complete *cox1 *fragment, transversions only**. Neighbour-Joining tree for the complete *cox1 *fragment (1009 sites) using Kimura 2-parameter distance and utilizing transversional substitutions only. 1000 bootstrap replicates have been performed. Bootstrap values below 50 are not shown. *B. africanus *group isolates are shown in red, *B. forskalii *in green, *B. reticulatus *in black and *B. truncatus/tropicu*s in blue. See Table 1 for origins of isolates.

**Figure 8:**
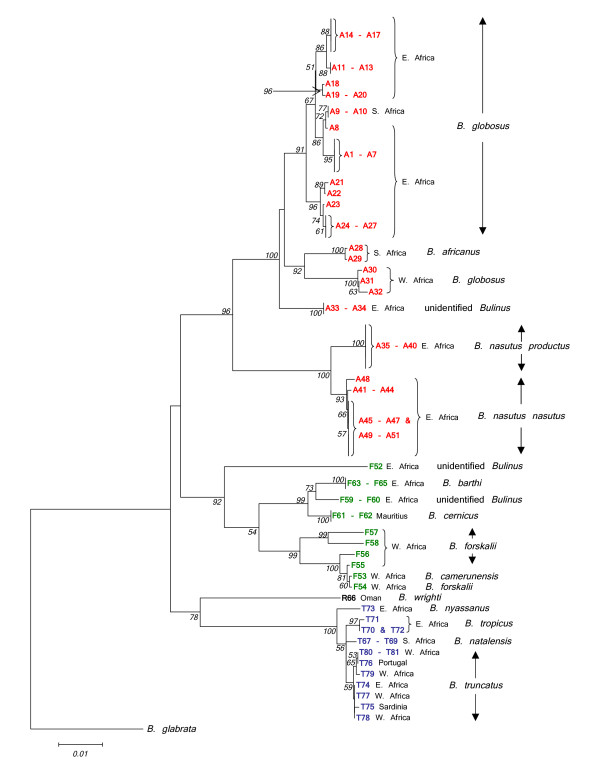
**Minimum Evolution tree for *Bulinus *isolates using the complete *cox1 *fragment, transversions only**. Minimum Evolution tree for the complete *cox1 *fragment (1009 sites) using Kimura 2-parameter distance and utilizing transversional substitutions only. 1000 bootstrap replicates have been performed. Bootstrap values below 50 are not shown. *B. africanus *group isolates are shown in red, *B. forskalii *in green, *B. reticulatus *in black and *B. truncatus/tropicu*s in blue. See Table 1 for origins of isolates.

The sequence of the mitochondrial *cox1 *gene within *Bulinus *was found to be highly variable. The nucleotide composition regarding this genus was AT rich (69.4%) which is in close agreement with previous work [12]. In order to test which areas of the *cox1 *amplicon gave the best phylogenetic signal, the fragment was analysed in its entirety, using the first 644 bp and also the final 387 bp. Stothard and Rollinson [9] and Jones *et al *[12] used the latter section of the *cox1 *sequence and this corresponds to the region encompassed by primers Asmit1 (AT1) and Asmit2 (AT2) shown in Fig. 2. The first 644 bp covers an area comparable to the sequence bounded by the Folmer primers LCO1490 (CO1) and HCO2198 (CO2) [18] and which has been used previously for barcoding studies [19]. Figure 4 shows three Neighbour-Joining trees generated for each region using both transitional and transversional substitutions. Resolution of the tree improves as progressively longer stretches of sequence are included in the analysis. However, saturation analysis of the 'Asmit' and 'Folmer' regions show that both are subject to saturation of transitional events suggesting that inclusion of transversional substitutions only in the calculation would present a more accurate estimate of isolate relationships within the genus. Figure 5 shows a graph where mean, pair-wise genetic distances of the two areas calculated from transversional substitutions only are compared with the corresponding distance matrix co-ordinates for the complete fragment. In this manner, the variability of genetic distance figures for the 'Asmit' and 'Folmer' regions relative to the complete fragment may be shown. For small genetic distance values correspondence is good between the three regions, however, as distances increase in size those associated with the 'Asmit' fragment tend to drift away from the corresponding complete sequence derived genetic distance values within the mid-range of the 'x' axis. Distances of the 'Folmer' region also exhibit a degree of variation when compared with those of the complete fragment but to a smaller extent. Additionally, a short span of genetic distance values between approximately 0.058 and 0.064 is entirely missing within the dataset. To a lesser degree this is reflected in a smaller region between 0.013 and 0.018. Figure 6 shows the same procedure undertaken for transitional substitutions where correspondence of distance values for the 'Asmit' and 'Folmer' areas again 'drifts' with increasing genetic distance relative to the complete fragment. However, whilst the overall number of pairs contributing to each distance figure is considerably less than in Fig. 5, the numerical variation of distance values present in the matrices is far greater, particularly between the ranges 0.035 to 0.077. Neighbour-Joining and Minimum Evolution trees were therefore generated for the dataset using transversions from the complete fragment sequence in order to obtain the maximum resolution possible. The resultant trees contained divisions corresponding to the four species groups (see Figs 7 &8).

## Discussion

### *B. africanus* species group

Classification within this complex is probably the most difficult of the four *Bulinus* snail species groups. The *cox1* sequence data for samples within this group revealed an extensive degree of genetic variation throughout the continent (see the designation 'A' in Figs 7 &8). There are three areas within the data set that may be used as reference points for interpretation of Figs 7 &8. The first is identified by sample code A23, a snail from Kinyasini, Unguja, which on the basis of previous work [8,9,20], represents the species known as *B. globosus*. The second are samples of *B. africanus*, from South Africa, namely, A28 and A29 and the third are those specimens labelled A35 to A40 and A41 to A51 representing *B. nasutus productus* and *B. nasutus nasutus*, respectively. The split of *B. nasutus* into two subspecies as recognised previously [2] may be clearly seen in Figs 7 &8 with *B. nasutus productus* being represented by samples from inland sites in Uganda and Tanzania and *B. nasutus nasutus* present in coastal Kenya and the Islands of Unguja, Pemba and Mafia. The molecular data support the view that these forms are closely related species and it would seem acceptable to consider them as *B. productus* and *B. nasutus*.

One of the most interesting facts to emerge is the division between samples of *B. globosus *from West and East Africa and the rather surprising finding that *B. africanus *has closer affinities to the West African *B. globosus *samples. *B. globosus *has the greatest geographical range of any member of this species group and it does seem that greater attention must be given to specific status and distribution. The *cox1 *barcode for *B. africanus *may be helpful in discriminating snails where species overlap especially when morphological differences are often difficult to determine, hence samples A9 and A10 identified as *B. globosus *can be distinguished from A28 and A29, *B. africanus*. The two species differ in the penis sheath, which is bigger in *B. africanus *being longer and/or thicker than the preputium [2] but such characters have been found unreliable for species discrimination [10].

Kenyan specimens from the Kisumu region used by Raahauge and Kristensen [10] have been included in the present study (A11 to A13 & A33). Utilizing RAPD profiles and PCR-RFLP results, these authors concluded that those samples labelled in Figs 7 &8 as A11 to A13 all appeared to be local variants of the same species and this conclusion is confirmed in the present analysis with all of the snails identified as *B. globosus*. In addition, they also showed that another sample screened in their study (ADC farm, Kisumu), labelled in Figs 7 &8 as A33, was different from the other Kisumu specimens and this has also been supported.

### *B. forskalii* species group

The group as a whole splits into two main sections, namely, snails from West Africa *i.e*. Cameroon, Bukina Faso, Niger, Senegal, São Tomé and Angola (F53 to F58) and those from the eastern side, East Kenya, Pemba, Mafia and Mauritius (F59 to 65). Additionally, isolate F52 from Uganda although sharing a common ancestor with East and West African *B. forskalii *group isolates, is quite distinct from the other species. However, only one specimen has been examined and more samples from this locality in Uganda are required for further study. The *its2 *tree showed F52 to have a closer relationship with East African members of the *B. forskalii *group. Within this group certain isolates are of known species and can be used as reference points, namely F58, which is *B. forskalii*, F61 and F62 both being *B. cernicus *from Mauritius and F63 to F65 from Pemba and Mafia considered by the authors as *B. barthi*.

Fewer isolates of the *B. forskalii *group have been tested and analysed as compared with the *B. africanus *group and so it might be imprudent to draw too many specific conclusions from the data. However, using fifteen allozyme loci from *B. camerunensis*, *B. forskalii *and *B. senegalensis*, Mimpfoundi & Greer [6] could find no differences between the two former species and suggested that the validity of *B. camerunensis *as a separate species might be open to question. Jones *et al *[12] used ITS1, RAPDs and *cox1 *to show that *B. camerunensis *clustered unequivocally with *B. forskalii *confirming that the taxonomic position of *B. camerunensis *is debatable. Only a single example of this species was available for our analysis (F53) although it came from the same area as that examined in Mimpfoundi's paper, namely, Lake Barombi, Kotto. The isolate shared genetic characteristics with *B. forskalii *from other West African countries such as Burkina Faso (F54) and Niger (F55), re-emphasising its questionable taxonomic status.

*Bulinus forskalii *from the island of São Tomé (F57) is responsible for the transmission of *S. guiniensis *and in this analysis grouped most closely to *B. forskalii *(F58) from Angola with both being well differentiated from other West African *B. forskalii*. Brown [21] was of the opinion that it was appropriate to identify the snails from São Tomé as extreme conchological variants of *B. forskalii*. Interestingly, in the analysis of Jones *et al *[12]*B. forskalii *from São Tomé clustered with *B. crystallinus *also from Angola. There is a need to sample and characterize more thoroughly *B. forskalii *group snails from Angola to assess their relationship with snails from São Tomé and West Africa as it appears that there may be more than one species involved.

*B. cernicus *from the island of Mauritius was once regarded as a form of *B. forskalii*, however, significant morphological differences with the latter were noted [2]. The current data reinforces the view that *B. cernicus *should be considered a separate species from both *B. forskalii *and *B. barthi*. Isolates F59 and F60 are also distinct from *B. forskalii*, *B. barthi *and *B. cernicus*. Stothard *et al *[22] sequenced the short 'Asmit' region for a snail collected from Mafia Island (SF369612). Although covering only a third of the current sequence for F59 and F60 the corresponding data match exactly and imply that this particular un-named *Bulinus *species is present in Pemba, Mafia and also East Kenya.

### *B. truncatus/tropicus* species group

Both Figs 7 and 8 confirm the position of *B. nyassanus *in the *B. truncatus/tropicus *complex and this species together with the cluster comprising *B. tropicus*, *B. natalensis *and *B. truncatus *appear to derive from a common ancestor. However, the conjecture by Nascetti & Bullini [5] that possible hybridization between *B. tropicus *and *B. natalensis *might have given rise to *B. truncatus *cannot be confirmed by the Minimum Evolution tree (Fig. 8). The observation by Brown [2] that there were indications of a "significant biological difference" between *B. tropicus *and *B. natalensis *is supported. The cluster representing *B. truncatus *in Figs 7 &8 has very short branch lengths implying that the hybridization event which Goldman *et al*. [3] suggested gave rise to this tetraploid is a relatively recent phenomenon.

### *B. reticulatus* group

There are only two recognised species within this group and it has only been possible to examine one of them, *B. wrighti*. This species has a characteristic *cox1 *sequence which positions the group close to the *B. truncatus/tropicus *complex.

### Barcoding

**Figure 9:**
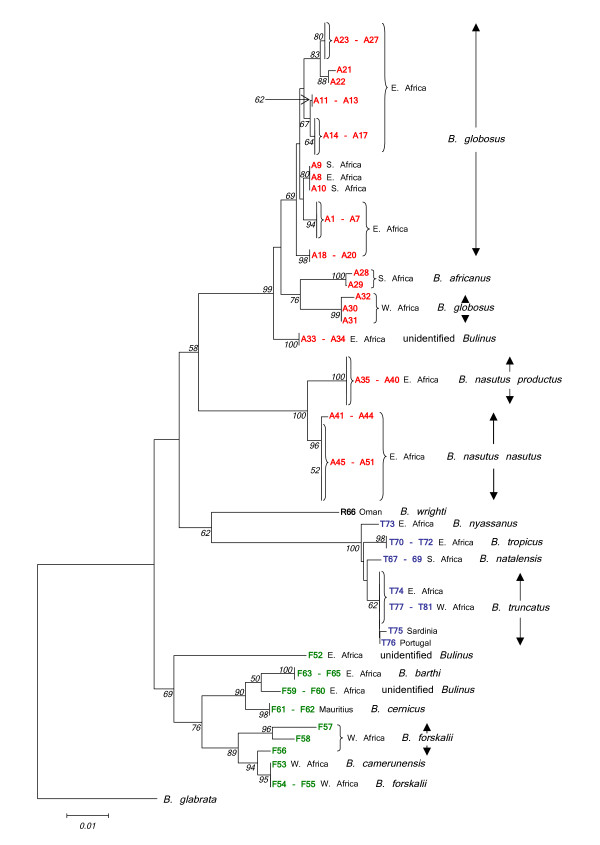
**Neighbour-Joining tree for *Bulinus *isolates using the 'Folmer' *cox1 *fragment, transversions only**. Neighbour-Joining tree using transversional substitutions only for the *cox1 *'Folmer' region. Calculation parameters are the same as for Figure 7. This region is proposed for potential use as a barcode. Bootstrap values below 50 are not shown. *B. africanus *group isolates are shown in red, *B. forskalii *in green, *B. reticulatus *in black and *B. truncatus/tropicu*s in blue. See Table 1 for origins of isolates.

The sequence information presented here is not a typical 'barcode' in so far as the sequence is longer than most barcodes which are usually around 650 bp in length [19,23,24]. Moreover, the generated PCR fragments have been amplified and sequenced using a wide variety of different primers due to the highly variable nature of the sequence. A pan-species group/isolate barcode in the usual 'sense' might be possible to locate but requires a common set of primers to be designed which will amplify all species within the genus for a particular *cox1 *region and that the area so determined mirrors the results generated by the current longer sequence. For this reason, a Neighbour-Joining tree (Fig. 9) has been generated for the area of *Bulinus cox1 *which corresponds approximately with the 'Folmer' barcode region. Agreement of Fig. 9 with Figs 7 &8 whilst not identical is very close and provides hope that this region could be used for barcoding *Bulinus *species in future.

The advantages [19] and disadvantages [25] over the use of barcoding, and the utilization of a single mitochondrial gene such as *cox1 *for identification and phylogenetic purposes have been the subject of considerable debate. It is accepted that a range of both nuclear and mitochondrial markers are required to provide a more accurate estimate of evolutionary history. However, a technique for routine screening which is relatively quick, cost effective and reproducible is essential given that resources are limited. The selected area of sequence from the *cox1 *gene appears to achieve this by forming a framework upon which a classification can begin to be constructed. Previous studies have used *cox1 *in the phylogenetic evaluation of *Bulinus*, namely, Stothard & Rollinson [9], Stothard *et al*. [11] and Jones *et al*. [12] and, in this respect, the current project is not unique, however, the sequences used in the present paper are three times the length of those previously employed and the range of *Bulinus *isolates/species much more extensive. The study is ongoing and undoubtedly as more taxa are acquired and sequences added to the database, the shape of the Neighbour-Joining and Minimum Evolution trees as shown in Figs 7 &8 will progressively alter and become more informative.

## Competing interests

The authors declare that they have no competing interests.

## Authors' contributions

RAK carried out the molecular work including nucleic acid extractions, PCRs, sequencing, sequence alignments, phylogenetic analysis and writing of the paper.

JRS collected material, analysed transitional saturation affects and compared transitional and transversional genetic distances with respect to different regions of the *cox1 *gene. He also aided in drafting the manuscript.

AME undertook some of the sequencing and produced Figures 5 and 6 following on from the work of JRS. He also helped draft the manuscript.

DR collected material, conceived the study, participated in its design and helped draft the manuscript.

All authors read and approved the final manuscript.

## Supplementary Material

Additional file 1 *cox1 *PCR fragments generated for sequencing and successful primers employed in both processes. Click here for file
