# Supplementary material for: Molecular characterization of freshwater snails in the genus Bulinus: a role for barcodes?
Source: Parasit Vectors. 2008 Jun 10;1:15. doi: 10.1186/1756-3305-1-15 (PMC2441610; doi:10.1186/1756-3305-1-15)
Supplement: Additional file 1 — cox1 PCR fragments generated for sequencing and successful primers employed in both processes. [file 1756-3305-1-15-S1.doc]

| **Label on tree** | **Amplified fragments** | **Successful sequencing primers** |
| --- | --- | --- |
| A1 | BC11 - AT2 | AT2, BC5, BC8, BC11, CO2 |
| A2 | BC5 - BAT2, BC12 - BC6 | BC5, BC6, BC12, BAT4 |
| A3 | BC5 - BAT4, BC11 - AT2, BC12 - BC6 | AT2, BC3, BC5, BC6, BC7, BC11, BC12, BAT4, CO2 |
| A4 | BC5 - BAT4, BC11 - AT2, BC12 - BC6 | AT2, BC3, BC5, BC7, BC11, BC12, BAT4, CO2 |
| A5 | BC5 - BAT4, BC11 - AT2, BC12 – BC6 | AT1, BC5, BC6, BC12, BAT4, CO2 |
| A6 | BC11 - AT2 | AT2, BC5, BC11, CO2 |
| A7 | BC5 - BAT4, BC11 - AT2, BC12 – BC6 | BC5, BC6, BC8, BC11, BC12, BAT4 |
| A8 | BC5 - BAT4, BC11 - AT2, BC11 - BC6, BC12 - BC6 | BC3, BC5, BC6, BC8, BC12, BAT4, CO2 |
| A9 | BC1-AT2, CO1-AT2 | AT1, AT2, BC1, BC3, BC5, CO1, CO2 |
| A10 | BC1-AT2, CO1-CO2 | AT2, BC1, BC5, CO2 |
| A11 | AT1 - AT2, BC1 - BC6 | AT1, AT2, BC1, BC2, BC3, BC4, BC5, BC6, CO2 |
| A12 | AT1 - AT2, BC1 - BC7, BC4 - BC6 | AT1, AT2, BC1, BC4, BC6, BC7 |
| A13 | AT1 - AT2, BC1 - BC6, BC1 - BC7, BC5 - BC6, BC4 - BC8 | AT1, AT2, BC1, BC2, BC5, BC6, BC7, BC8 |
| A14 | AT1 - AT2, BC1 - BC7, BC4 - BC6 | AT1, AT2, BC1, BC4, BC6, BC7, CO2 |
| A15 | AT1 - AT2, BC1 - BC7, BC4 - BC6 | AT1, AT2, BC1, BC4, BC6, BC7 |
| A16 | BC1 - AT2 | AT2, BC1, BC5, BC6 |
| A17 | BC1 - AT2, BC5 - BAT4, BC12 - BC6 | AT2, BC1, BC5,BC6, BC8, BC12, BAT4 |
| A18 | AT1 - AT2, CO1 - AT2, CO1 - CO2 | AT1, AT2, BC5, BC6, CO1, CO2 |
| A19 | BC1 - BC6, BC5 - BC14, CO1 - BC7 | BC1, BC2, BC3, BC5, BC7, BC8, BC12, BC14, CO1, CO2 |
| A20 | BC1 - BC6, BC5 - BC14, CO1 - BC7, CO1 - BC10 | BC1, BC2, BC5, BC7, BC8, BC12, BC14, |
| A21 | BC5 - BC14, BC12 - BC6 | BC3, BC5, BC6, BC12, BC14 |
| A22 | BC5 - BC14, BC12 - BC6 | BC3, BC5, BC6, BC12, BC14 |
| A23 | AT1 - AT2, BC1 - CO2, BC1 - BC6 | AT1, AT2, BC1, BC2, BC3, BC4, BC5, BC6, CO2 |
| A24 | BC5 - BC14, BC12 - BC6 | BC3, BC5, BC7, BC8, BC12, BC14 |
| A25 | BC5 - BC14, BC12 - BC6 | BC3, BC5, BC7, BC8, BC12, BC14, CO2 |
| A26 | BC1 - BC6, BC5 - BC14 | BC1, BC3, BC5, BC6, BC12, BC14, CO1 |
| A27 | BC1 - BC6, BC5 - BC14 | BC1, BC5, BC6, BC12, BC14, CO1 |
| A28 | BC1 - AT2 | AT1, AT2, BC1, BC5, CO2 |
| A29 | BC1 - AT2 | AT1, AT2, BC1, BC5, CO2 |
| A30 | AT1 – AT2, BC1 – BC6 | AT1, AT2, BC1, BC5, BC6, CO2 |
| A31 | AT1 - AT2, BC5 - BC6, CO1 - AT2, CO1 - C02, | AT1, AT2, BC5, BC6, CO1, CO2 |
| A32 | CO1 - AT2 | AT1, AT2, BC5, BC6, CO1,C02 |
| A33 | AT1 - AT2, BC5 - BC6, CO1 - AT2, CO1 - CO2 | AT1, AT2, BC5, BC6, CO1, CO2, |
| A34 | AT1 - AT2, BC1 - BC6, BC5 - BC6 | AT1, AT2, BC1, BC2, BC3, BC4, BC5, CO2 |
| A35 | CO1 - AT2 | AT2, BC5, BC6, CO1, CO2 |
| A36 | CO1 - AT2 | AT1, AT2, BC1, BC5, BC6, BC9, CO1, CO2 |
| A37 | AT1 - AT2, BC1 - BC7, BC4 - BC6 | AT1, AT2, BC1, BC4, BC6, BC7 |
| A38 | BC12 - BC6, BC5 - BAT4 | BC3, BC5, BC6, BC7, BC12, BAT4 |
| A39 | AT1 - AT2, BC1 - BC7, BC4 - BC6 | AT1, AT2, BC1, BC4, BC6, BC7 |
| A40 | AT1 - AT2, CO1 - AT2, CO1 - CO2 | AT1, AT2, BC5, BC6, CO1, CO2 |
| A41 | BC12 - AT2 | AT2, BC5, BC12, CO2 |
| A42 | BC12 - AT2 | AT1, AT2, BC5, BC8, BC12, CO2 |
| A43 | BC12 - AT2 | AT2, BC5, BC12, CO2 |
| A44 | BC12 - AT2 | AT2, BC5, BC12, CO2 |
| A45 | AT1 - AT2, BC5 - BC6, CO1 - CO2 | AT1, AT2, BC5, BC6, CO1, CO2 |
| A46 | BC12 - AT2 | AT2, BC3, BC5, BC6, BC7, BC8, BC10, BC12 |
| A47 | BC12 - AT2 | AT2, BC5, BC6, BC7, BC8, BC10, BC12, CO2 |
| A48 | BC9 - BAT4, BC12 - BC6 | AT1, BC6, BC9, BC12, BAT4, CO2 |
| A49 | BC5 - BC14, BC12 - BC6 | BC3, BC5, BC6, BC12, BC14 |
| A50 | BC5 - BC14, BC12 - BC6 | BC3, BC5, BC6, BC12, BC14 |
| A51 | BC5 - BC14, BC12 - BC6 | BC3, BC5, BC6, BC12, BC14 |
| F52 | CO1N3 - AT2 | AT1, AT2, BC5, BC6, CO1N3, CO2 |
| F53 | BC1 - AT2 | AT1, AT2, BC1, BC5, BC6, BC7, BC8, CO2 |
| F54 | AT1 - AT2, BC1 - BC7, BC4 - BC6 | AT1, AT2, BC1, BC4, BC6, BC7 |
| F55 | AT1 - AT2, BC1 - BC7, BC4 - BC6 | AT1, AT2, BC1, BC4, BC6, BC7 |
| F56 | BC1 - AT2 | AT1, AT2, BC1, BC5, BC8, CO2 |
| F57 | BC1 - AT2 | AT1, AT2, BC1, BC5, BC7, BC8, CO2 |
| F58 | BC1 - AT2 | AT1, AT2, BC1, BC5, BC7, BC8, CO2 |
| F59 | AT1 - BC14, BC5 - BC14, BC5 - BAT4, BC12 - BC6 | AT1, BC6, BC12, BC14, CO2 |
| F60 | BC5 - BC14, BC12 - BC6 | BC3, BC5, BC6, BC12, BC14 |
| F61 | BC1 - AT2, BC5 - AT2, BC1 - CO2 | AT2, BC1, BC5, CO2 |
| F62 | BC1 - AT2 | AT1, AT2, BC1, BC5, BC7, BC8, CO2 |
| F63 | CO1 - AT2 | AT1, AT2, BC5, BC6, CO1, CO2 |
| F64 | CO1 - AT2 | AT2, BC1, BC5, BC6, CO1, CO2 |
| F65 | CO1 - AT2 | AT2, BC1, BC5, BC6, CO1, CO2 |
| R66 | BC1 - AT2 | AT2, BC1, BC5, BC7, BC8, CO2 |
| T67 | BC1 - AT2 | AT1, AT2, BC1, BC5, BC6, BC7, BC8, CO2 |
| T68 | BC5 - BC14, BC12 - BC6 | BC3, BC5, BC6, BC7, BC12, BC14 |
| T69 | BC5 - BC14, BC12 - BC6 | AT1, BC3, BC5, BC6, BC7, BC12, BC14 |
| T70 | BC5 - BAT4, BC12 - BC6, BC13 - BC6 | BC3, BC5, BC6, BC12, BAT3, BAT4 |
| T71 | BC5 - BAT4, BC12 - BC6 | BC3, BC5, BC6, BC7, BC12, BAT4 |
| T72 | BC5 - BAT4, BC12 - BC6, BC13 - BC6 | BC3, BC5,BC6, BC12, BAT4, CO2 |
| T73 | BC5 – BC14, BC5 – BAT4, BC12 – BC6 | AT1, BC5, BC6, BC12, BC14, BAT3, BAT4, CO2 |
| T74 | AT1 - AT2, BC1 - BC7, BC4 - BC6 | AT1, AT2, BC1, BC4, BC6, BC7 |
| T75 | BC1 - AT2, BC1 - CO2, BC5 - AT2, CO1 - BC3 | AT2, BC1, BC3, BC5, CO1, CO2 |
| T76 | BC1 - AT2, BC1 - CO2, BC5 - AT2 | AT2, BC1, BC3, BC5, BC7, CO1, CO2 |
| T77 | CO1 - AT2 | AT2, BC5, BC6, CO1, C02 |
| T78 | CO1 - AT2 | AT1, AT2, BC5, BC6, CO1, C02 |
| T79 | AT1 - AT2, BC1 - BC7, BC4 - BC6 | AT1, AT2, BC1, BC4, BC6, BC7 |
| T80 | CO1 - AT2 | AT2, BC5, CO1, CO2, |
| T81 | AT1 - AT2, BC1 - BC7, BC4 - BC6 | AT1, AT2, BC1, BC4, BC6, BC7 |
